# Pharmacokinetic/Toxicity Properties of the New Anti-Staphylococcal Lead Compound SK-03-92

**DOI:** 10.3390/antibiotics4040617

**Published:** 2015-11-24

**Authors:** William R. Schwan, Jill M. Kolesar, M. Shahjahan Kabir, Edmund J. Elder, Jeffrey B. Williams, Rachel Minerath, James M. Cook, Christopher M. Witzigmann, Aaron Monte, Tricia Flaherty

**Affiliations:** 1 Department of Microbiology, University of Wisconsin-La Crosse, 1725 State St., La Crosse, WI 54601, USA; E-Mails: minerath.rach@uwlax.edu (R.M.); flaherty.tric@uwlax.edu (T.F.); 2 Emerging Technology Center for Pharmaceutical Development, University of Wisconsin-La Crosse, 1725 State St., La Crosse, WI 54601, USA; E-Mail: amonte@uwlax.edu; 3 Carbone Comprehensive Cancer Center, University of Wisconsin-Madison, Madison, WI 53706, USA; E-Mail: jill.kolesar@wisc.edu; 4 Department of Chemistry and Biochemistry, University of Wisconsin-Milwaukee, Milwaukee, WI 53211, USA; E-Mails: mkabir@uwm.edu (M.S.K.); capncook@uwm.edu (J.M.C.); witzigm2@uwm.edu (C.M.W.); 5 Zeeh Pharmaceutical Experiment Station, University of Wisconsin-Madison, Madison, WI 53705, USA; E-Mails: edmund.elder@wisc.edu (E.J.E.); jbwz17@gmail.com (J.B.W.); 6 Department of Chemistry and Biochemistry, University of Wisconsin-La Crosse, 1725 State St., La Crosse, WI 54601, USA

**Keywords:** *Staphylococcus*, pharmacokinetics, safety testing, drug formulation

## Abstract

Because of the potential of a new anti-staphylococcal lead compound SK-03-92 as a topical antibiotic, a patch, or an orally active drug, we sought to determine its safety profile and oral bioavailability. SK-03-92 had a high IC_50_ (125 μg/mL) *in vitro* against several mammalian cell lines, and mice injected intraperiteonally at the highest dose did not exhibit gross toxicity (e.g., altered gait, ungroomed, significant weight loss). Single dose (100 μg/g) pharmacokinetic (PK) analysis with formulated SK-03-92 showed that peak plasma concentration (1.64 μg/mL) was achieved at 20–30 min. Oral relative bioavailability was 8%, and the drug half-life was 20–30 min, demonstrating that SK-03-92 is likely not a candidate for oral delivery. Five-day and two-week PK analyses demonstrated that SK-03-92 plasma levels were low. Multi-dose analysis showed no gross adverse effects to the mice and a SK-03-92 peak plasma concentration of 2.12 μg/mL with the presence of significant concentrations of breakdown products 15 min after dosing. SK-03-92 appeared to be very safe based on tissue culture and mouse gross toxicity determinations, but the peak plasma concentration suggests that a pro-drug of SK-03-92 or preparation of analogs of SK-03-92 with greater bioavailability and longer half-lives are warranted.

## 1. Introduction

*Staphylococcus aureus* is the leading cause of skin infections in the United States [1]. Nearly 60% of the *S. aureus* clinical isolates are methicillin-resistant *S. aureus* (MRSA). Although skin infections caused by MRSA remain high, hospital-associated invasive MRSA infections have decreased [2]. In addition to methicillin resistance, MRSA strains continue to evolve resistance to other antibiotics [3].

**Figure 1:**
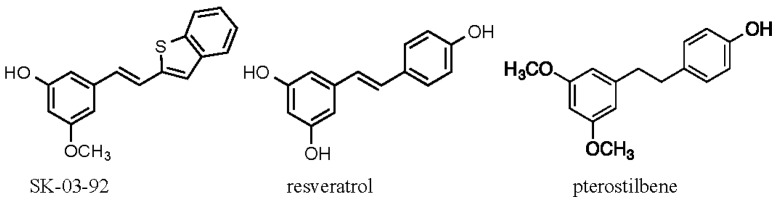
Structures of SK-03-92, resveratrol, and pterostilbene.

Because of this growing problem with antibiotic resistance in *S. aureus*, new antibiotics with novel mechanisms of action are needed. *S. aureus* is one of the ESKAPE pathogens (*Enterococcus faecium*, *Staphylococcus aureus*, *Klebsiella pneumoniae*, *Acinetobacter baumannii*, *Pseudomonas aeruginosa*, and *Enterobacter* species), which are targeted by the 10 × '20 initiative to get 10 new, safe and effective antibiotics approved by 2020 [4]. As part of this initiative, we have directed our research efforts at identifying anti-infective natural products in plants and mushrooms. Previously, we isolated (*E*)-3-hydroxy-5-methoxystilbene from the leaves of the shrub, *Comptonia peregrina* (L.) Coulter (“sweet fern”), which was used medicinally in the past by Native North Americans [5]. A structural analog of this natural product, (*E*)-3-(2-(benzo[*b*]thiophen-2-yl)vinyl)-5-methoxyphenol (given the code name, SK-03-92), was synthesized (Figure 1), and the MIC_50_ and MIC_90_ values against *S. aureus* (including MRSA and VISA strains) were determined to be 1 μg/mL and 2 μg/mL, respectively [6]. In this study, we ran pharmacokinetic (PK) analyses to determine if SK-03-92 was still a viable lead compound. We discovered that SK-03-92 had poor solubility characteristics.

## 2. Results and Discussion

### 2.1. Physical Properties of SK-03-92

In an effort to advance SK-03-92 through preclinical testing, the Advanced Chemistry Development (ACD) software program version 8.08 was used to predict the physical properties of this agent. SK-03-92 (chemical formula C_17_H_14_O_2_S, with a molecular weight of 282.36 g/mol) had a predicted pKa value of 9.25 and a cLogP value of 6.50, demonstrating its relatively high lipophilicity and low solubility in aqueous solutions through a pH range of 2.0–10.0 (1.63 × 10^−3^ to 2.51 × 10^−4^ μg/mL). The core scaffold of SK-03-92 is a stilbene, and stilbene-like compounds (e.g., resveratrol; Figure 1) have shown poor solubility in aqueous solutions [7].

### 2.2. Determining the Solubility of SK-03-92 in Other Solutions

Because SK-03-92 had low solubility in an aqueous solution, and solubilizing the drug in DMSO would be inappropriate for oral or intravenous (IV) administration to humans, alternative vehicles were examined. The solubility of SK-03-92 was determined using high performance liquid chromatography (HPLC). Solubility of SK-03-92 in PBS was <0.0001 mg/mL, but the solubility in other suitable vehicles ranged from 4.6 mg/mL for 20% Solutol HS 15/80% PBS to 9.2 mg/mL for PEG 400. The Solutol HS 15/PBS solution was chosen for further development of a dosing solution due to the potential for use in both IV and oral administration [8].

### 2.3. Safety/Cytotoxicity Testing of SK-03-92

To determine the approximate safety of SK-03-92 against tissue culture cells, an MTT [3-(4,5-dimethylthiazol-2-yl)-2,5-diphenyltetrazolium bromide] assay [9] was performed. The IC_50_ of SK-03-92 was shown to be 125 μg/mL for all four tissue culture cell lines (J774A.1, U937, 292, and T24), generating a promising relatively high therapeutic index (IC_50_/MIC) of 62.5 for 90% of the *S. aureus* strains.

To determine if SK-03-92 was safe for use *in vivo*, an escalating dosing scheme [10] in mice (5, 50, 300, and 2000 mg/kg) was used. None of the mice that received a single dose of highest drug concentration (2000 mg/kg) IP displayed any adverse effects over a two-week time frame (e.g., altered gait, ungroomed, significant weight loss), demonstrating that SK-03-92 was non-toxic at this highest dose. After initial dosing at 2000 mg/kg, a repeat dose of 2000 mg/kg was administered a week later, and again, no overt toxicity was observed for the animals. High doses of the related compounds, pterostilbene (Figure 1) and resveratrol, have been shown to be non-toxic in mice [11], rats [12], and humans [13].

### 2.4. Single Dose PK Analysis of SK-03-92

A single dose of SK-03-92 was given too mice to assess single dose PK parameters. The observed area under the plasma concentration-time curve (AUCINF_obs), maximum plasma concentration (C_max_), time to achieve C_max_ (T_max_), oral clearance of drug observed (Cl_F_obs), volume of distribution of drug observed (Vz_F_obs), and half-life were calculated. Intraperitoneally administered SK-03-92 had a half-life of 22.46 min, a T_max_ of 30 min, a C_max_ of 1.64 μg/mL, a Cl_F_obs of 1.46 mL/min/g, a Vz_F_obs of 47.71 mL/g, and a AUCINF_obs of 70.15 min·mg/mL (Table 1). The T_max_ of 30 min when given ip and 22 min when dosed orally demonstrated rapid absorption of SK-03-92 in line with the rapid absorption of other stilbenoid drugs [13,14]. The maximal plasma concentration (C_max_) was somewhat low for orally administered SK-03-92 (370 ng/mL), but was still detectable and was approximately 25% of the C_max_ observed with ip-administered drug (1.64 μg/mL). The C_max_ and AUC_0-inf_ values for SK-03-92 fall between the values reported for resveratrol and pterostilbene [15]. After achieving the C_max_, the SK-03-92 plasma concentrations dropped to negligible levels 75 min after oral gavage, which was in line with the PK results that have been observed using other stilbenoid drugs [13,15,16,17,18,19]. The relatively short half-life of SK-03-92 (22 min for ip administered, 30 min for orally administered) suggests that administration more than once a day would be needed to treat a *S. aureus* infection. The concentration of SK-03-92 reached a high of 1.641 μg/mL after 30 min and dropped to 0.149 μg/mL within 90 min of ip injection. All of the mice looked healthy, and no overt toxicity (e.g., excessive weight loss, ungroomed, altered gait) was observed, mimicking the mouse safety study above.

**Table 1:** Summary of pharmacokinetic (PK) parameters by route and schedule.

Route	Half-Life (min)	T_max_^c^ (min)	C_max_^c^ (μg/mL)	Cl_F_obs ^c^ (mL/min/g)	Vz_F_obs ^c^ (mL/g)	AUCINF_obs ^c^ (min·μg/mL)
ip ^a^	22.46 ± 17.81	30.00 ± 0.00	1.64 ± 0.59	1.46 ± 0.26	47.71 ± 33.25	70.15 ± 12.76
oral ^b^	30.40 ± 17.81	21.43 ± 11.80	0.37 ± 0.35	21.00 ± 6.71	810.67 ± 173.87	5.14 ± 1.87

^a^ Mean ± standard deviation from three mice tested; ^b^ Mean ± standard deviation from three mice tested; ^c^ Tmax (time to achieve Cmax), Cmax (maxium plasma concentration), Cl_F-obs (oral clearance of drug observed), Vz_F-obs (volume of distribution of drug observed), and AUCINF_obs (area under the plasma concentration-time curve observed).

### 2.5. Relative Bioavailability of SK-03-92

To determine the relative bioavailability of the SK-03-92 lead compound, 100 μg/g of Solutol HS 15/PBS formulated SK-03-92 was administered orally to the mice. After 15 min, 0.740 μg/g was detected in the mouse plasma, and by 30 min that level had dropped to 0.126 μg/g. All of the mice looked healthy, and no toxicity signs were observed. Relative bioavailability (AUC_oral_/AUC_IP_) was approximately 8%, indicating that oral delivery of the drug would not provide therapeutic concentrations for treating *S. aureus* infections. Other studies that have examined stilbenoid compounds have also observed low to very low bioavailability for those agents [7,13,15,16,17,18,19].

### 2.6. Multi-Dose Effects and Protein Analyses of SK-03-92

The single dose PK analysis showed the SK-03-92 lead compound was safe, but the plasma concentration was somewhat low. Multi-dose experiments were performed to assess adverse effects associated with chronic dosing as well as pharmacokinetics. Given that adverse effects do not correlate linearily with plasma concentrations, animals could experience adverse effects when plasma concentrations were undetectable with repeated dosing. Pharmacokinetic evaluations were performed to determine if changes in pharmacokinetic profiles with chronic dosing (e.g., reduced clearance) were associated with adverse effects.

To determine how multiple doses of SK-03-92 affected the health of the mice as well as the PK parameters, an extended five-day PK study was undertaken. Plasma concentrations ranged from a high of 0.086 μg/mL after 15 min to a low of 0.027 μg/mL after 75 min. In the PK study extended to two-weeks following oral administration, the plasma SK-03-92 concentrations ranged from 0.144 μg/mL after 15 min to 0.01 μg/mL after 75 min. On average, the treated mice lost about 1.5% of their weight after two-days, but after one week, the weight of treated mice increased on average by 5.16% on day 9 and went up further to 9.75% by day 12. Two doses of SK-03-92 given four hours apart for three days achieved a maximum SK-03-92 plasma concentration of only 2.12 μg/mL.

### 2.7. Protein Binding by SK-03-92

The degree of protein binding by SK-03-92 in plasma was also determined. It was found that 84.4% of the SK-03-92 drug bound to plasma proteins. Thus, most of the SK-03-92 lead compound bound to plasma proteins that could affect its bioavailability.

This was the first study to examine the PK properties of a new lead compound labeled SK-03-92. Other studies have examined the PK properties of stilbenoid-based compounds, but none of these other studies have done PK analysis with the expressed goal of using a drug with a stilbene scaffold to treat bacterial infections. The absence of overt symptoms in mice following dosing with SK-03-92 suggests that SK-03-92 may have a very good safety margin. High concentrations of SK-03-92 given daily to mice did not appear to have gross adverse effects on the mice, which is in line with animal studies done using resveratrol and pterostilbene [11,15,16]. Both the SK-03-92 ip and oral administration pharmacokinetic profiles were low; likewise, low plasma concentrations of resveratrol (<1 μg/mL) and pterostilbene (<8 μg/mL) have been observed in animals [15]. The maximum SK-03-92 plasma concentration in the mice was only 2.12 μg/mL. Several properties of SK-03-92 probably contribute to its low plasma/tissue concentrations in mice. Aqueous solubility appeared to be one of the barriers. Stilbene compounds are hydrophobic and generally insoluble in aqueous solutions [7,20,21]. The use of a Solutol HS 15/PBS solution helped improve the solubility of SK-03-92, but the solubility was still less than 10%. Another property tied to the low plasma/tissue availability of SK-03-92 is its high level of plasma protein binding (84.4%). Our previous *in vitro* study demonstrated a much higher MIC against *Streptococcus pneumoniae* [6], and this was probably due to SK-03-92 binding to the proteins found in the fetal calf serum added to the wells of the microtiter plate. A myriad of proteins present in the bloodstream and tissues would reduce the effective concentration of SK-03-92. Lastly, SK-03-92 exhibited a relatively short half-life in the mice, breaking down into metabolic byproducts within 15 min after ip or oral delivery. Resveratrol and pterostilbene also have short half-lives in animal plasma (15–60 min and 120 min, respectively) with substantial accumulations of metabolic byproducts [7,12,15,16].

New drugs with novel mechanisms of action are needed to keep abreast of antibiotic resistance in *S. aureus*, one of the ESKAPE pathogens. The safety profile of SK-03-92 is highly encouraging. Although the oral bioavailability of SK-03-92 may hinder use as an oral stand-alone treatment for *S. aureus* infections, the opportunity to employ it against drug resistant strains of MRSA topically or embedded in a patch still exists. Further work on the structure activity relationships of this class of compound to improve potency, duration of action and water solubility are underway. We are also analyzing microarrays and performing additional assays to elucidate the mechanism(s) of action of SK-03-92. Furthermore, we are currently assessing whether SK-03-92 has a synergistic effect when co-administered with clinically useful antimicrobials such as oxacillin, clindamycin, and vancomycin. If the half-life and bioavailability problems can be solved, an analog of SK-03-92 may have a future in treating *S. aureus* skin and soft tissue infections.

## 3. Experimental Section

### 3.1. Synthesis of SK-03-92

A gram of SK-03-92 was synthesized, as previously described [6].

### 3.2. Physical Property Assessment

An Advanced Chemistry Development (ACD) software program version 8.08 was used to predict the physical properties of SK-03-92.

### 3.3. Mice

The Institutional Animal Care and Use Committees at the University of Wisconsin-La Crosse and the University of Wisconsin-Madison approved the study design and the animal handling protocols of this pharmacokinetic study. The protocol number for UW-Madison was M1732 with a start date of 23 April 2009, whereas the UW-La Crosse protocol was 3–8 with a start date of 26 March 2008. Either female BALB/c (NCI) or Swiss Webster (Harlan) mice 5–9 weeks old were used in this study.

### 3.4. Cytotoxicity Assays

Initial *in vitro* safety testing of SK-03-92 was performed with an MTT [3-(4,5-dimethylthiazol-2-yl)-2,5-diphenyltetrazolium bromide] assay (9, American Type Culture Collection) for several tissue culture cell lines (murine monocytic J774A.1, human monocytic U937, human kidney epithelial 293, and human bladder epithelial T24) using mitomycin C (Sigma, St. Louis, MO, USA) and DMSO alone as positive and negative controls, respectively. Assays were done a total of three times per tissue culture cell line.

### 3.5. Initial Safety Testing in Mice

Three female 5–9 week old Swiss Webster mice (Harlan) per drug concentration were injected intraperitoneally (ip) with increasing concentrations of SK-03-92: 5, 50, 300, and 2000 mg/kg according to OECD [10]. The lowest concentration of drug was used first. Overt toxicity (e.g., altered gait, ungroomed, significant weight loss) was monitored. If two of three mice showed no overt toxicity, then the next dose of drug was given.

### 3.6. HPLC Assay Development

An HPLC method was developed on a Halo C18 4.6 × 1.0 mm, 2.7 μm column (MAC-MOD Analytical Inc., Chadds Ford, PA, USA) for the detection and quantitation of SK-03-92. The method was developed using the information obtained from a scouting gradient of 5%–100% acetonitrile over 35 min. A UV spectrum of SK-03-92 was obtained from the scouting gradient and the observed maximum for SK-03-92 was 335 nm, with a local maximum of 224 nm also observed. The 335 nm maximum wavelength was used for the quantitation of SK-03-92, and the 224 nm local maximum wavelength was used to monitor for possible formation of degradation products not visible at the 335 nm wavelength. Flow rate was 0.750 mL/min and injection volume was 10 μL. The mobile phase was 62% acetonitrile (Fisher, Hanover Park, IL, USA):38% water (Milli-Q UV plus) and the isocratic program was 62% acetonitrile:38% water for 20 min. Acquisition time was 10 min and room temperature retention time was 5.4 min. Standards of SK-03-92 were prepared in acetonitrile at concentrations of 0.05–0.005 mg/mL.

### 3.7. Single Dose PK Assay

An initial single dose PK experiment was performed using 100 μg/g of formulated SK-03-92 administered ip to twenty-one female, 5–6 week old BALB/c mice (7 per cohort for 3 cohorts, NCI) under parameters established previously [22]. Three animals were used as a control group and received blank vehicle, whereas three mice per time point received 100 μg/g SK-03-92 ip and were sacrificed with blood collected at the 15, 30, 45, 60, 75, and 90 min time points by warming the tails of the mice in warm water, lancing the lateral saphenous tail vein, and collecting the blood (up to 150 μL) into microvette tubes (Sarstedt) containing EDTA or terminal bleed into vacucontainers containing EDTA as an anti-coagulant. Plasma was separated from packed blood cells by centrifugation (600× *g*) and stored at −70 °C until needed for HPLC analysis. Samples were analyzed with a Shimadzu Prominence HPLC system (Kyoto, Japan) with a Halo C18 column (MAC-MOD Analytical Inc., Chadds Ford, PA, USA). A mobile phase of 62% acetonitrile/38% water, isocratic program of 62% acetonitrile for 20 min, and flow rate of 0.75 mL/min with an injection volume of 10 μL and a retention time of 5.4 min were used. The standard curve for SK-03-92 was linear from 0.0098 to 2.5 μg/mL in plasma. Each chromatogram was collected and integrated to estimate the peak area of analyte by EZ Chrom^®^ software (version.1.0, Agilent Technologies Inc., Santa Clara, CA, USA). The ratio of peak area of SK-03-92 was calculated to estimate the drug concentration(s) using a pre-built calibration curve. Major pharmacokinetic parameters were calculated using the noncompartmental analysis module of WinNonlin^®^ (version 5.1, Pharsight Corp., Princeton, NJ, USA) under sparsely sampling mode [22].

### 3.8. Bioavailability Assay

A relative bioavailability study was performed using 100 μg/g of formulated SK-03-92 administered orally to 6 female 5–6 week old BALB/c mice (NCI) with three animals as the control group receiving blank vehicle by the oral route. Three mice per time point had blood collected at 15 and 30 min after administration.

### 3.9. Multi-Dose Analysis

First, a five-day PK study with SK-03-92 orally administered to female 5-6 week old BALB/c mice was conducted. Three mice received 300 μL of blank vehicle by oral administration. Doses of 100 μg/g SK-03-92 were given orally each day in the vehicle. Blood was collected from three mice at time points 15, 30, 45, 60, 75, and 90 min after administration. Body weight was monitored every day before SK-03-92 administration. Plasma was prepared and stored at −70 °C until analyzed by HPLC. In an extension of this PK study to two-weeks following oral administration of SK-03-92, six mice were dosed in the test (SK-03-92) arm and six vehicle control mice in the other arm. Lastly, a multi-dosing PK analysis was done using two 51-μg/g doses of SK-03-92 per day (given four hours apart) over the course of three days in five mice.

### 3.10. Protein Binding Assay

Plasma collected from EDTA treated blood from six SK-03-92 dosed mice was added to methyl *tert*-butyl ether (MTBE) to precipitate the proteins. The precipitated proteins were separated from the remainder of the plasma by centrifugation (14,000× *g* for 20 min) through a Microcon 10K filter (Millipore) and dried under a stream of nitrogen gas. The dried product was suspended in 62% acetonitrile/38% ddH_2_O and the AUC was quantified and correlated with the initial concentration of SK-03-92 present in the plasma.

## 4. Conclusions

SK-03-92 lead compound displayed a very good safety profile, however, the PK results indicate that further refinement of the SK-03-92 structure is warranted to achieve a therapeutic plasma concentration.
